# Proposing Circular Economy Ecosystem for Chinese SMEs: A Systematic Review

**DOI:** 10.3390/ijerph18052395

**Published:** 2021-03-01

**Authors:** Zhejun Min, Sukanlaya Sawang, Robbert A. Kivits

**Affiliations:** 1 Faculty of Business and Law, Coventry University, Coventry CV1 5FB, UK; minz@uni.coventry.ac.uk (Z.M.); ac5611@coventry.ac.uk (R.A.K.); 2 QUT Business School, Queensland University of Technology, Brisbane 4000, Australia

**Keywords:** circular economy (CE), SMEs, circular economy (CE) ecosystem

## Abstract

Circular economy (CE) has attracted so much attention around the world as it can contribute to the balance between economic development and environmental sustainability, to address the increasing critical resources scarcity and environmental issues. Small and medium-sized enterprises (SMEs) in China are a major driving force in the economy with 43 million SMEs in 2020. Most of them maintain the traditional economic development method of “take–use”, without considering the environment. This has caused great harm to the environment and resource availability. Therefore, Chinese SMEs must adopt CE in their business to address this issue. The current study aims to explore the key barriers (lack of time, lack of human resources and finance) and enablers (e.g., network, innovation, and reputation) for Chinese SMEs CE adoption. The current study employs a systematic review approach with thematic analysis to identify the internal and external barriers and enablers of CE adoption among SMEs. Moreover, a CE ecosystem has been proposed for identifying the key actors in the CE system, which will be beneficial for policy-makers to take into account when drafting and adjusting CE laws and regulations.

## 1. Introduction

In recent years, the circular economy (CE) has gained much attention globally since it has been regarded as a promising approach to tackle the problems between resource usage and environmental impacts [1]. There have been many countries adopting the CE principles to their laws and national development strategies, amongst others are China, Germany, and Japan [2]. According to previous research, small and medium-sized companies (SMEs) are less often the object of investigation on their environmental involvement compared to large enterprises [3]. In general, SMEs are often considered as failing to take into consideration environmental sustainability due to their lower numbers of participation in sustainable business practices under the large enterprises’ standards [4]. However, it is undeniable that SMEs have a constructive and vital influence on the national economy, by greatly stimulating the growth of personal income, employment, and export expansion [5]. In 2018, Chinese SMEs represented 99.8% of total businesses and provide 79.4% job positions to employees nationwide [6,7]. They also contribute to 60% of GDP and more than 50% of taxes [8]. Because of the size of the SME industry sector in China, it is an essential part of the market to target for CE adoption, as even a minor percentage increase in adoption has a significant impact. Therefore, SME CE adoption in China becomes essential and beneficial for the whole of CE promotion in China.

The current study aims to systematically review the important enablers and barriers, using a proposed CE ecosystem concept as a framework for the review. The current study aims to answer the question, “What are the important enablers and barriers for Chinese SMEs to adopt CE principal in their business”? The current study seeks to make two theoretical contributions to the literature. First, this study views the CE practice of Chinese SMEs by adopting a systematic literature review method. Since most research focuses on CE promotion and implementation at a national level and large-scale enterprises’ involvement in China, little attention is paid to the role of Chinese SMEs in the CE, and factors that influence its development. Therefore, this research fills the academic gap in this field. Second, the proposed CE ecosystem framework is integrated by CE system and ecosystem, which will be beneficial for discovering different actors in the closed-loop ecological, economic system, to better cooperate with the completion of circular sustainable development.

The current study also has a significant practical contribution. First, it allows policymakers to see the influencing factors for Chinese SMEs to adopt CE and design a comprehensive and effective CE development plan for its future implementation. Second, the CE ecosystem framework enables other key actors to find their own positions and understand their relationship with other actors to collaborate.

This paper is structured as follows: Section 2 discusses the CE framework with relevant literature within the Chinese context. Section 3 details secondary data used and the methodology. Qualitative results and discussions are provided in Section 4. Section 5 concludes the overall research findings and recommendations. Finally Section 6 and Section 7 discuss research limitation and future research.

## 2. Literature Review

### 2.1. The Implementation of Circular Economy (CE) in China

China’s rapid economic development over the last thirty years has led to the rapid destruction of the environment, which is detrimental to people’s health and to social development [9,10]. Consequently, building an environment-friendly and resource-saving society is the urgent and long-term strategic plan for China [11,12]. CE forms an important part of this long-term strategic plan. Yong [13] proposed that the evolution of China’s CE can be divided into three stages. 

The first period, from 1998 up to 2002, is generally regarded as the *conceptual introduction stage*. Chinese scholars firstly introduced the concept named as recycling economy or circular economy (CE) in 1998. The State Environmental Protection Administration of China (SEPA) found the principles of recycling economy conducive to environmental protection, so it promoted this concept nationwide by supporting CE research, carrying out pilot projects of municipal and provincial cleaner production and proposing to the State Council to develop CE. In 2002, the importance of CE implementation in China was mentioned in the Second Global Environment Facility. 

From 2003 to 2005 is the *national government decision-making stage*. Since 2003, the Central Government proposed the scientific development concepts where the CE was believed as critical to long-term economic strategies. In 2004, the National Development and Reform Commission (NDRC) was appointed to be fully in charge of CE implementation and promotion, which enabled CE to become China’s basic national policy. In 2005, the State Council made a plan for CE and proposed to integrate CE into the 11th Five-year Planning Outline for National Economic and Social Development. 

Post 2005 can be seen as the *nationwide pilot demonstration and CE development stage*. In October 2005, the first list of pilot entities was issued by the NDRC, which included 56 enterprises, 13 industrial parks, seven provinces, five cities, and one town. In December 2007, the second list was announced, and the total number of pilot entities increased to 178. Since 1 January 2009, the first CE law named as circular economy (CE) Promotion Law of the People’s Republic of China was enacted. It is indicated that the CE implementation was actively and comprehensively promoted top-down within China. In 2013, the Development Strategy and the Immediate Action Plan of circular economy (CE) was issued for making strategic planning and specific arrangements towards CE development, ultimately to comprehensively improve the level of ecological civilisation [14].

China’s CE practices can be demonstrated from three levels, macro-level, meso-level, and micro-level (Table 1). 

The macro-level mainly refers to city or regional scale, and focuses on the development of eco-cities, eco-municipalities, or eco-provinces via policies and regulations [15]. applying the Reduce, Reuse, and Recycle (3R) principles and local situations such as regional characteristics and hefty polluting enterprises, the city’s infrastructure and industrial layout can be redesigned and rearranged to promote both sustainable production and consumption activities, to achieve a recycling-oriented society [9]. 

The meso-level mainly centres on industrial clusters or eco-industrial parks where sustainable designs can improve resource efficiency and product up-gradation to achieve industrial symbiosis; meanwhile, the green supply chain inside ensures and improves the resource circulation within the region [10,16]. Yong [13] demonstrates that two types of eco-industrial parks in China include new parks and existing parks integrating 3R principles. The CE activities are implemented in two ways: developing eco-industrial chain among enterprises in the park and share infrastructural systems for all enterprises as standard supplies. Taking Tianjin Economic Development Area and Suzhou Industrial Park as examples, these show how the enterprises inside have been implementing waste exchange and enhancing symbiotic relationships among different firms [10].

At the micro-level, enterprises in production, such as factories, agricultural product producers and other manufactures, are stimulated to apply cleaner production and eco-design of manufacturing plants, waste management to their real-life work practices [9]. Eco-design (eco-innovation) can be interpreted as the design (innovation) incorporated with environmental concerns and is adopted in their production process and products [15]. Additionally, it has been proven that enterprises with a mature environmental management system are more willing to implement CE as they have recognized the importance of environmental improvement, and how this is right for their reputation among customers and allows cost-saving [17]. 

In brief, at the micro-level, enterprises need to permeate environmental protection thinking into their products and management, such as environmental design, clean products and the like. At the meso-level, enterprises in the ecological park should exchange resources through cooperation and create environmental protection supply chains to achieve industrial symbiosis. At the macro-level, the formulation of national and regional policies and regulations can significantly promote the CE development at the meso and micro levels.

### 2.2. Small and Medium Enterprises’ Adoption in China

According to the National Law of the People’s Republic of China on the Promotion of Small and Medium-sized Enterprises (2002), SMEs in China refers to enterprises under various ownerships established in China’s territory. Their scales in production and operation are small and medium-sized, which are in line with the industrial and the State requirements, and offer more job opportunities. The classification criteria of SMEs, formulated by the department under the State Council, are the number of employees, volume of sale, total assets, and business types. More specifically, these are enterprises with fewer than 2000 employees, an annual turnover less than 300 million RMB, or total assets equal or less than 400 million RMB [7].

China’s SMEs play a vital role in contributing to the country’s social, economic, and industrial development. According to Zeng, et al. [18], more than 90% of the manufacturing enterprises in China were composed by SMEs in 2010, which brought enormous profits, but also caused severe environmental pollution. In addition, SMEs in printing and dyeing, food processing, and chemical industries all had similar issues, gradually polluted the surrounding environment and severely affecting the neighbouring residents’ health [7]. The main reasons for this impact can be traced back to the fact that SMEs were using outdated and obsolete machinery and technology, and untrained and inexperienced labourers. This is often due to insufficient financial resources and limited environmental management, causing less efficient arrangement and usage of resources [19]. It was reported by the China Environmental Protection Administration that approximately 50% of the environmental pollution in China is caused by SMEs, and 80% of the 30 million SMEs produce environmental pollution in one way or another [18]. Therefore, the adoption of CE by SMEs is crucial in the successful promotion and implementation of CE in China. 

In order to improve and alleviate the heavy energy consumption and environmental pollution, the Chinese government has been taking action since 1996, and more than 150,000 polluting SMEs, were either closed, suspended, merged or transformed. However, these measures only work temporarily [7]. Although the Cleaner Production Promotion Law (CPPL) of China took effect in 2003, their scope was limited. Zhang, et al. [20] state that still now, larger firms, as opposed to SMEs, are more active in the CE implementation, under pressure from governmental regulations, community participation and market demand. For Chinese SMEs, there are many barriers to adopting CE as a part of their business operations. Main barirers include; lack of capital, lack of government support, lack of information, lack of technical and technological know-how, lack of support from networks, limited company environmental culture, and perceived increased administrative burden [21]. Despite this situation, Prieto-Sandoval et al. (2018) indicate that academics, policy-makers, practitioners, government, and institutions can be considered as the key actors who can facilitate CE adoption among SMEs in China. However, as the current literature on enablers and barriers of Chinese SMEs’ CE adoption is far from satisfactory, there may be more influencing factors. Therefore, the current study will introduce the CE ecosystem framework to explore other key actors affecting CE adoption among SMEs in China.

### 2.3. Circular Economy Ecosystem

CE proposes a closed-loop economy and aims to minimise waste and emissions, and maximise the usage of resources via its four different loops, such as product-life extension, redistribution/reuse, remanufacturing, and recycling [22,23]. To close these loops it is very important to establish collaboration among various actors in the CE system [24]. In addition, CE requires a whole-system design, a transformation of production and consumption systems, cross-sector collaboration, a shift from supply chains to value networks, and life-cycle thinking [25]. The stability of the circularity in the CE system can be changed by the interactions among actors, products, components and materials [25]. Therefore, higher degree cooperation among different actors is extremely vital [26].

The CE Ecosystem can be regarded as the conceptual framework for exploring the influencing actors in the CE process. Unfortunately there is limited literature and research on CE Ecosystems. Mainly the actors in CE are identified as policy-makers, investors, academics and educators, designers, consumers and users, brands or companies, manufacturers, and material experts [27]. The current study thus adopts the CE Ecosystem as a guideline for thematic analysis, answering the proposed research question, “What are the important enablers and barriers for Chinese SMEs to adopt CE principals into their businesses”? 

## 3. Method

According to Petticrew and Roberts [28], systematic literature reviews aim to explore and cope with research activities within a specified field via scientific approaches. In order to present the barriers and facilitators of Chinese SMEs’ CE adoption, this paper applies a systematic review based on the evaluation of relevant publications, and overlays a thematic analysis for further explorations [29]. In order to ensure and maintain transparency and replicability, this paper follows the principles of systematic reviews; planning, conducting, and reporting, as outlined by Tranfield, et al. [30].

According to Jones, Coviello and Tang [29], the planning stage should include a literature search method, detailed inclusion and exclusion criteria for article selection and analytical process. Therefore, firstly, suitable search terms are defined for selecting the relevant literature. The key phrases are set, such as “circular economy (CE) practices in China”, “SMEs and circular economy (CE)”, “circular economy ecosystem”, “sustainable technologies”, “sustainable processes”, “sustainable business”, “green business”, “environmental management and SMEs”, and “environmental management and SMEs in China”. Secondly, the inclusion and exclusion criteria are applied in the selection process to ensure transparency and accuracy [31]. The inclusion criteria are (a) SMEs context; (b) China as a study region; (c) peer review articles. 

The in-depth literature search is conducted primarily in three databases: ScienceDirect, Business Source Complete, and ProQuest. For the sake of improving the searching efficiency and effectiveness, there are several exclusion standards to assess. For each database, the key phrases are applied within the title, abstract and keywords. The research publication time covers the last 10 years between 2010 and 2020, and the language is restricted to English only. The publication type is confined as peer-reviewed journal articles. As a result, 551 articles were at the time identified as potentially relevant to the topic. After applying all inclusion criteria above, the final review sample was reduced to 31 articles. These articles explicitly focus on the influencing factors for SMEs’ CE adaption, which enriches the actors of the CE ecosystem. As a result, the barriers and enablers for Chinese SMEs’ CE adoption will be identified for theoretical and practical usages. In order to illustrate the distinct features of current research on Chinese SMEs’ CE adoption circumstance, an inductive, interpretation-based approach of theme identification is conducted [29]. 

The next stage is the thematic analysis of all the chosen articles. After re-reading all the selected articles, the initial codes for themes are targeted and concluded in Microsoft Excel which is a useful tool for theme identification. The initial codes for themes from each article are put together for comparison and further modifications. To sum up, the whole analysis process follows the rules of validating coding by returning to the full-text article and comparing it to other articles in the sample. 

After the first round coding, the first-order themes for each article which are related to the research questions are captured. Then, all the first-order themes are mapped, grouped and sorted. Based on it, the second-order themes are concluded for a higher-order level. The second-order themes are then grouped into thematic areas on an even higher level of abstraction in terms of meaning, purpose and coding of the articles. After it, the first-order and the second-order themes can be cross-checked. In the end, themes and associated articles are assigned to one of two main taxonomies focusing on the role of Chinese SMEs’ critical enablers and barriers in this process, respectively.

## 4. Results and Discussion

According to the results of the thematic analysis, the 31 reviewed articles can be classified into two main themes, (i) Barriers for SMEs’ CE Adoption (*n* = 26) and (ii) Enablers for SMEs’ CE Adoption (*n* = 29). The selected articles comprise thematic areas that were developed from second-order themes and, as the smallest entity, first-order themes. The latter part demonstrates the key actors in the CE ecosystem. The thematic areas and themes are concluded in Table 2 and Table 3.

### 4.1. Category 1: Barriers for SMEs’ CE Adoption

The barriers for SMEs’ adoption of CE can be viewed as internal and external barriers (Table 2). The internal barriers are classified as *resources* (e.g., lack of time) and *capabilities* (e.g., lack of human creativity). The next section discusses the emerging themes in details.

#### 4.1.1. Internal Barriers

The second-order themes, internal barriers, include two first-order themes: resources and capabilities, which deeply explores the internal influencing factors of SMEs. 

In terms of *resources*, most of SMEs lack capital and investment from investors, due to their small turnover, which leads SMEs to pay more attention to their own profitability and seek survival and future development [32,33]. Further, there are great expense related to acquiring CE equipment, and implementation of distribution planning, inventory management, and training of personnel, which hinder CE implementation for SMEs with limited funding [21,34,35]. Secondly, learning and upskilling about CE takes a long time, and to design and integrate CE with SMEs operations and development strategies, to implement CE and to get successful outcomes is not in line with the status quo of SMEs and the current economic climate as SMEs are looking for short-term gains [36]. In addition, most SMEs claim that a lack of suitable and attainable technology and sufficient technical resources has largely restricted their attempts to implement CE [35,37]. In terms of lack of human resources, technical specialists are highly required in CE adoption and implementation. Availability of such specialists can greatly eliminate the burden of administrative tasks such as monitoring and reporting on CE data, and they can improve the whole CE performance [21,38]. 

Regarding *capabilities*, first, most SMEs lack knowledge and/or creativity in organisational culture due to China’s complex bureaucratic system. For this reason many business owners or managers have not realised the importance of, or comprehend the essence and vision of CE adoption [35]. For example, many business leaders only promote, and participate in CE practices because of reimbursable grant programmes [32]. Second, according to Wang [33], the life expectancy of Chinese SMEs is short, normally 3 to 5 years, which impedes the continuity and consistency of CE implementation and development. 

#### 4.1.2. External Barriers

The second-order theme external barriers emerged from the first-order themes are *Political Aspect, Economic Aspect, Social Aspect* and *Legal Aspect*. This group primarily examines the external factors which constrain the SMEs’ CE adoption.

First, from the *political aspect*, lack of government support, exemplified by unclear and outdated guidelines on CE for SMEs, training courses for workers, less funding opportunities and effective taxation policies, has been noted as a large issue for SMEs’ CE adoption [21,39]. The formulation of CE guidelines and some regulations is more suitable for large companies rather than most SMEs [40]. In addition, bureaucratic difficulty in administration makes it harder for SMEs to implement CE in their business. For instance, the response time on CE inquiries from the government and institutional agencies are always uncertain, which appears to delay and adversely impacts on SMEs when they try to accommodate CE [40]. 

From the *economic perspective*, the operation of SMEs and their decisions to implement CE are associated with the national economic system and national funding mechanism [31]. As the current economic system still maintains the profit-driven characteristic, the traditional economy has not been changed radically, especially among SMEs [34]. The current market structure is not friendly to SMEs which do not have enough funding to adopt CE into their business, compared to larger enterprises [41]. 

Thirdly, from the *view of the society*, the public or customer awareness in CE is not strong enough to stimulate SMEs to comply with public or market needs, which is the catalyst for conversion to the CE [21]. Lastly, from the *legal perspective*, as Garcés-Ayerbe, Rivera-Torres, Suárez-Perales and Leyva-de la Hiz [38] state; CE laws, policies, and regulations in CE should be set clearly and easily to understand and implement, as they are the guidance determining the status of SMEs CE adoption. Moreover, SMEs feel more pressure than large corporations in legislation, because it has strict regulations on SMEs’ systems and qualifications [3]. 

While these barriers have been identified, it is also recognized that these barriers can gradually be overcome given enough time. Essential to this process is the understanding of the key actors involved in the whole process of the CE ecosystem.

### 4.2. Category 2: Enablers for SMEs’ CE Adoption

The second group of papers are focussed on the Enablers for SMEs’ CE adoption. These enablers can be classified into Internal Enablers and External Enablers. As the barriers and enablers for SMEs’ CE adoption are interrelated factors, the chosen papers are mainly focused on these themes (Table 3). 

#### 4.2.1. Internal Enablers

The third-order Internal Enablers are composed of second-order themes; *Resources* and *Capabilities*, and first-order themes; *Network, Innovation, Reputation* and *Finance*.

Drawing from *resource perspective*, the network is considered as the facilitator for knowledge sharing and coordination with various stakeholders [42,43]. First, strategic partnerships built between large corporations and SMEs can be an effective approach. This is because SMEs are the critical power of innovation and complex problem solvers, while large corporations have economic strength and resources, which can mutually support each other and create collective values [15,32,34]. Within the cooperation, knowledge and resource sharing can encourage CE adoption and improve enterprises’ environmental management [44]. Second, with the continuous increase of corporate cooperation, industrial clusters have gradually formed and achieved industrial symbiosis. Industrial clusters can collectively encourage SMEs to adopt CE, such as local sourcing, cooperation via supply chain and infrastructure sharing, can ensure the product quality, reduce the time of the entire industrial process, and boost enterprises’ involvement and innovation, which can shape their competitiveness in the market [15,34,45]. Thirdly, stakeholder involvement is viewed as a crucial element on SMEs’ CE adoption, which can be classified into internal and external stakeholders [35]. Specifically, internal stakeholders include owners, managers and employees, where the comprehensive engagements positively contribute to the CE practices; by contrast, external stakeholders indicate customers, society and government, where customers are the critical driver of CE adoption [35]. Customers’ and competitors’ requirements become the strong engine to push SMEs to take part in CE practices [18]. The network covers a broad range of actors, namely as manufacturers, suppliers, large corporations, business owners etc., which enables SMEs to consider all the actors’ requirements and make the CE ecosystem active. 

Next, *capability aspect* entails Innovation, Reputation and Finance. Firstly, innovation is the main driving force for SMEs to achieve organisational transformation and CE adoption. Innovation can be divided into three levels, business model innovation, organisational innovation and product innovation. Business model innovation is primarily supported by cooperation with large companies, which can compensate for each other’s deficiency to promote CE practices [34]. The organisational innovation includes organisational structure, operation, and culture, which may significantly alter the survival status and development mode of SMEs [46]. Regarding organisational structure innovation, the re-division of department settings and responsibilities is critical. For example, SMEs can assign a person or a department to be in charge of the adoption and implementation of the CE, and also they will conduct CE training for the other departments in the company, which will help the company meet various regulatory requirements and realise the systematisation in the CE [47]. 

In terms of *innovation* towards CE, the core is that SMEs should mainly focus on their upstream and downstream business to capture resources [46]. Meanwhile, SMEs still need to continuously leverage their CE tools and adjust its operation process to streamlined one which not only improves resource utilisation, but also meet the global standards [35]. Such operation management innovation can also improve stakeholders’ satisfaction and enhance organisational governance. Organisational culture innovation refers to the mindset, habits and attitudes towards CE of the business owners, managers and employees, which are assumed as a vital part for CE transition in SMEs [21]. It has been pointed out that business owners and managers’ attitudes and behaviour towards environmental management, company leadership, are the enablers of CE practices; their involvement highly improves employees’ motivation and work efficiency in CE [21,36]. 

#### 4.2.2. External Enablers

The third-order theme External Enablers derived from the second-order themes *Political Aspect, Social Aspect, Environmental Aspect,* and *Legal Aspect*.

In the *political perspective*, firstly, government incentive, including tax reduction, subsidies, compensation, venture capital, investment, other financial facilitation and chances to participate in government’s programmes, motivates SMEs to adopt and implement CE the most [18,48]. It enables CE policies, regulations, and knowledge spread effectively and rapidly, and also stimulates the initiative of SMEs [44]. 

From the *social aspect*, media exposure is the fastest and most efficient way for the public to acquire CE and push SMEs to conduct CE practices. By increasing media exposure, public awareness of CE and sustainable development can be quickly cultivated, which facilitates the development of CE practices at all levels of society. SMEs exposed for violating or failing CE laws and regulations by media will receive pressure from the public and even get a penalty from local government. Community and social requirements are the suggestions and complain to the SMEs, which may harm the neighbouring environment [18]. 

In terms of the *environmental aspect*, the CE adoption and practice can enable local environment recover bit by bit and benefit the community and society, which serves the driving force for enterprises to contribute themselves to the local community and improve their reputation [17]. 

Lastly, in the *legal aspect*, the enactment of CE laws and regulations strengthens the binding force to the SMEs, which drives them to make decisions and implement CE [41,49]. Compliance with legislation is identified as the primary influencing enabler of CE or environmental enhancement [34]. For example, EU Regulations, such as CE Policies, REACH, RoHS, urge enterprises to integrate their products and services with CE requirements and make them in line with the global standard [34]. China’s Cleaner Production Promotion Law in 2003 clarifies the duties and penalties towards enterprises which fail to meet the law [18].

## 5. Conclusions

The current study is conducted via a systematic literature review to address the research questions, the key barriers and enablers of SMEs’ CE adoption in China. On the basis of the results, the key barriers and enablers for Chinese SMEs’ CE adoption are identified and concluded. Taking Chinese SMEs as individuals, its CE adoption practice can be explored into two sides, its internal and external influencing factors. The internal side of SMEs is focused on its resources and capabilities, while its external sides are on political, economic, social, environmental, and legal aspects.

### 5.1. Internal Influencing Factors

With respect to resources, the barriers for most SMEs to adopt CE are limited funds, lack of investment, inadequate time, lack of technology, technical resources, and technical specialists. Limited finance can only support SMEs’ daily business operation and may end up with a limited turnover, which leads to less attractive to investors. Moreover, the whole process of CE acquisition, adoption, and implementation need substantial time [50], which is not realistic for SMEs to complete it on their own. Likewise, CE adoption requires a high standard of technologies, technical resources, and specialists to supervise its implementation, which is both time-consuming and money-consuming and may be futile in the end.

In order to solve these problems and get SMEs better involved in CE practices, the network is the critical facilitator. The network includes collaboration with large enterprises, industrial clusters, and stakeholder involvement. Strategic partnerships with large enterprises enable SMEs to use their own advantages to exchange resources with large companies or upstream and downstream companies, thereby making up for the lack of funds, technology, and resources. Industrial clusters can facilitate SMEs to grasp the latest trend in the industry, understand the policies, regulations, and technologies of CE, to optimise resources enjoy public facilities to reduce expenditures, and ultimately achieve industrial symbiosis. Stakeholder involvement can inspire each stakeholder, such as customer, supplier, manufacturer and the like, to understand CE and proactively participate in the CE practices, which is conducive to stimulating SMEs to conduct CE practices with initiatives. In terms of SMEs’ capabilities, lack of human creativity and short life expectancy is shown as the barriers. Lack of human creativity leads to organisational culture and leadership without considering the environment or CE as part of their strategy into their business. SMEs’ short survival time makes it harder to adopt and implement CE. 

Given the above, innovation, reputation, and finance are the major enabling factors to address them. First, innovation covers business model innovation, organisational innovation and product innovation. Business model innovation, such as cooperation with large companies, can enable SMEs to gain resource and technological advantages while improving their business chains and opening up new markets. Organisational innovation adjusts the organisational structure, such as adding CE specialist or department, changing the organisational operation mode to focus on stakeholders collaboration, and promoting CE culture. Product innovation is obtained by integrating eco-design into the existing products, which adds values for the products and shape the competitiveness of the company, to enhance the viability of SMEs. Second, adopting CE to their business can reflect the corporate social responsibility and set a good example of compliance with laws and regulations, contributing to keeping talents and improving prestige. Third, although the investment of CE adoption is vast at the beginning, from a long-term consideration, it can necessarily reduce costs and continuously achieve values.

### 5.2. External Influencing Factors

From the political perspective, lack of government support in clear guidance for SMEs, training, funding supports, and inefficient bureaucratic administration greatly affected the participation of SMEs [51]. Therefore, it has been suggested that government incentives, such as tax reduction, subsidies, and compensation, are the key driver. From the economic aspect, it mainly presents a barrier for SMEs’ CE adoption. Since the current economic climate, national economic system and market structure remain profit-oriented, most SMEs still conduct their business in the traditional, take–use, economical approach. From the social point, currently, public awareness in CE is still not enough, which turn out to be the obstacle of implementation. The enablers are media and community requirements. Media exposure is shown as the most effective and efficient way to push SMEs’ CE adoption. 

In the view of the environment, the recovery of the local environment has been seen as the enabling factor for SMEs, which wants to build a positive social image and benefit its community. Lastly, from the legal side, the compulsion of CE laws and regulations is an essential stimulating factor for SMEs to introduce CE. However, in some regulations, the information is not clear, and the actual situation of SMEs is not considered, which also causes SMEs to avoid the implementation of CE to some extent.

### 5.3. CE Ecosystem Key Actors

As described, after identifying the key barriers and enablers, the key actors in CE ecosystem can be enriched and concluded, namely, as business owners, managers, employees, technical specialist, media, regulator, institution agencies, and banks. These actors in the CE ecosystem framework will be conductive for probing the CE implementations.

Although there are many pieces of research studying CE implementation and SMEs’ CE practices, most of them concentrate on the European countries’ SMEs, China’s national or industrial level CE practices. It is evident to find out from the current literature that there is a gap in exploring how SMEs introduce and implement CE in their business operation in China and what are the obstacles and driving forces for them. 

In order to handle these issues, the current study tries to set up a CE ecosystem framework where the key actors in CE system can be identified to promote the future CE adoption and implementation in China, which contributes to the literature in this field. There are several practical implications. From the government and policy maker’s view, the barriers and enablers identified and actors involved for SMEs can be considered when making and adjusting policies, as SMEs are the primary driving force of GDP. The regulations and laws should consider SMEs practical situations and their downsides compared with large enterprises. From the academic perspective, the CE ecosystem can not only be used in SMEs’ CE adoption but also can be the guidance for other directions in the CE system.

## 6. Limitations of the Study

Although this research has both theoretical and practical values, there are still several limitations that should be considered. First, this research is only based on peer-reviewed journal articles. Second, the research topic is focused on Chinese’ SMEs CE adoption, which is quite broad. Different industries, locations, firm sizes, and so forth, will have different barriers and enablers. Moreover, the level of CE adoption is not specified. Thirdly, there are only three searching engines (ProQuest, Business Source Complete, and ScienceDirect) used in the research, which may exclude some other valuable journals from the other searching engine. Fourthly, this research is a systematic review based on other researchers’ views rather than the actual visits and interviews, which may have different understandings and interpretations.

## 7. Future Research

The future research in this topic can be further examined within specific industries within a country. Cross country comparison of SMEs can also advance our knowledge of CE ecosystem, which can be impacted by local regulation or global phenomena such as climate change. The research method will combine systematic review and interviews, which can be tested through a theoretical and practical scenario.

## Figures and Tables

**Table 1:** Three Levels of Circular Economy (CE) Practices in China (adapted from [10,14]).

Areas	Macro (Province, Region and Cities)	Meso (Inter Firms)	Micro (Enterprises)
Production	Eco-city, Eco-municipality, and Eco-province	Eco-industrial park	Cleaner production
Consumption	Renting service	Environmental friendly park	Green purchase & consumption
Waste Management	Urban symbiosis	Waste trade market Industrial symbiosis	Product reuse and recycle system
Design	Environmental friendly design	Environmental friendly design	Eco-design

**Table 2:** Barriers for Small and medium-sized enterprises (SMEs’) CE Adoption.

Thematic Area	Second-Order Theme	First-Order Theme	Theme Description	Key Actors
Barriers for SMEs’ CE Adoption	Internal Barriers	Resources	➢ Lack of time ➢ Lack of capital and investment ➢ Lack of technology and technical expertise ➢ Lack of human resources	Investor, banking institutions, Suppliers, manufacturers, Academia/Professionals, manager, owner, technical specialist
		Capabilities	➢ Lack of human creativity ➢ SMEs’ short survival time;	manager, business owner, employees
	External Barriers	Political Aspect	➢ Lack of government support ➢ Bureaucratic difficulty in administration;	Government, Policy makers, Institutional Agencies
		Economic Aspect	➢ National economic system and national funding mechanisms ➢ Market structure	Government and banking institutions
		Social Aspect	➢ Public awareness	The public, customers
		Legal Aspect	➢ Unclear and complex regulations and standards ➢ Legislation pressure for SMEs	Government, Policy makers, SMEs

**Table 3:** Enablers for SMEs’ CE Adoption.

Thematic Area	Third-Order Theme	Second-Order Theme	First-Order Theme	Theme Description	Key Actors
Enablers for SMEs’ CE Adoption	Internal Enablers	Resources	Network	➢ Strategic partnerships built between large corporations and SMEs ➢ Industrial clusters ➢ Stakeholder Involvement	Manufacturers, suppliers, large corporations, business owners, managers, employees, customers, government and society
		Capabilities	Innovation	➢ Business model innovation ➢ Organisational innovation	SMEs, large corporations, business owners, managers, employees, CE experts
			Reputation	➢ SMEs’ social prestige	SMEs, employees, society
			Finance	➢ Profitability ➢ Continuous value capture	SMEs, suppliers, manufactures
	External Enablers	Political Aspect	-	➢ Government incentive	Government, institutional agencies, SMEs
		Social Aspect	-	➢ Public awareness ➢ Media exposure ➢ Community requirements	Media, the public, SMEs
		Environment Aspect	-	➢ Recovery of local environment	SMEs, community
		Legal Aspect	-	➢ CE Laws and regulations	Government, Regulator, Policy Maker

## Data Availability

Data is contained within the article.
